# Monostotic fibrous dysplasia in the proximal tibial epiphysis: a case report

**DOI:** 10.1186/1752-1947-8-452

**Published:** 2014-12-20

**Authors:** Ji-Yong Gwark, Jin-Hoon Jeong, Sun-Chul Hwang, Dae-Cheol Nam, Jeong-Hee Lee, Jae-Boem Na, Dong-Hee Kim

**Affiliations:** 28 Department of Orthopaedic Surgery, Research Institute of Clinical Medicine, Gyeongsang National University School of Medicine and Hospital, 15 Jinju-daero 816 beon-gil, Jinju, 660-750 South Korea; 29 Department of Pathology, Gyeongsang National University School of Medicine and Hospital, Jinju, Korea; 30 Department of Radiology, Gyeongsang National University School of Medicine and Hospital, Jinju, Korea

**Keywords:** Bone tumor, Epiphysis, Fibrous dysplasia

## Abstract

**Introduction:**

Fibrous dysplasia is one of many well-known disorders in which there is a defect in the remodeling process of immature bone to mature into lamellar bone, and it often exists in metaphyseal and diaphyseal parts of the long bone. In this report, we describe a rare case where fibrous dysplasia was found only in the proximal part of the epiphysis of the tibia without other bony lesions.

**Case presentation:**

A 14-year-old Asian girl was referred to our hospital after slipping down with pain on the left knee. A radiograph showed an abnormal finding of a central radiolucent lesion with a marginal sclerotic border near the proximal tibial spine. A magnetic resonance image showed the lesion at low signal intensity on a T1-weighted image and at high signal intensity on a T2-weighted image. The biopsy results led us to conclude that the lesion was a fibrous dysplasia.

**Conclusion:**

If an abnormal lesion on the epiphysis, especially in long bones, is detected on a radiograph, several differential diagnoses can be made. Although fibrous dysplasia is usually not encountered as an epiphyseal lesion, it is important to incorporate all the clinical, radiographic and pathologic features to diagnose monostotic fibrous dysplasia when the lesion is located at the epiphyseal location.

## Introduction

The term *fibrous dysplasia* (FD) was coined by Lichtenstein in 1938 to describe a subset of benign bone tumors manifesting in childhood or early adult life. FD has a tendency of predominantly unilateral involvement and a prolonged clinical course characterized by pain, deformity and pathologic fracture of the affected bones [1]. FD can be manifested as a single lesion (monostotic) or as multiple lesions (oligostotic or polyostotic). According to Ippolito and colleagues, in monostotic FD, the most commonly appearing site is on the femur. Tibia, humerus, rib, clavicle and craniofacial skeleton are the next in order of frequency [2]. McCune-Albright syndrome is known as a combined condition consisting of polyostotic bone involvement, precocious puberty, hyperthyroidism and café au lait cutaneous macules [3, 4]. Mazabraud syndrome is associated with single or multiple intramuscular myxomas in monostotic or polyostotic form [5]. In this report, we describe a very rare case of a patient with monostotic FD involved solely in the epiphysis of proximal tibia.

## Case presentation

**Figure 1:**
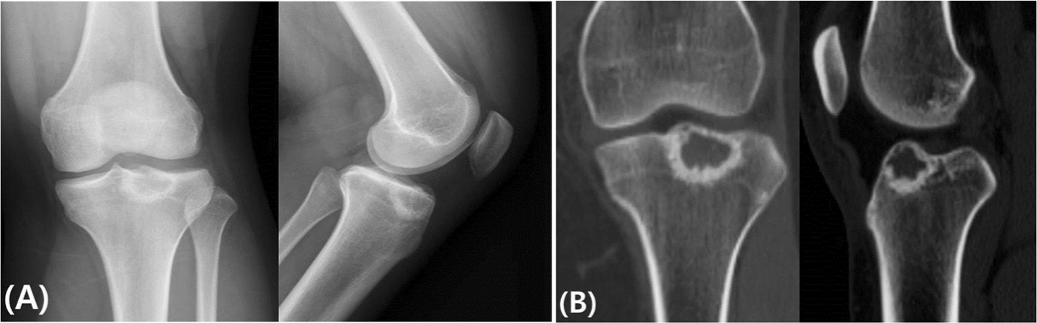
**Plain radiographic images and computed tomograhic scan. (A)** Anteroposterior (left) and lateral (right) radiographs show the proximal tibial epiphyseal osteolytic lesion with marginal sclerosis and a matrix with a ground glass appearance and without periosteal reaction. **(B)** Coronal (left) and sagittal (right) computed tomographic scans show the absence of cartilage matrix and dense marginal sclerosis without cortical disruption.

**Figure 2:**
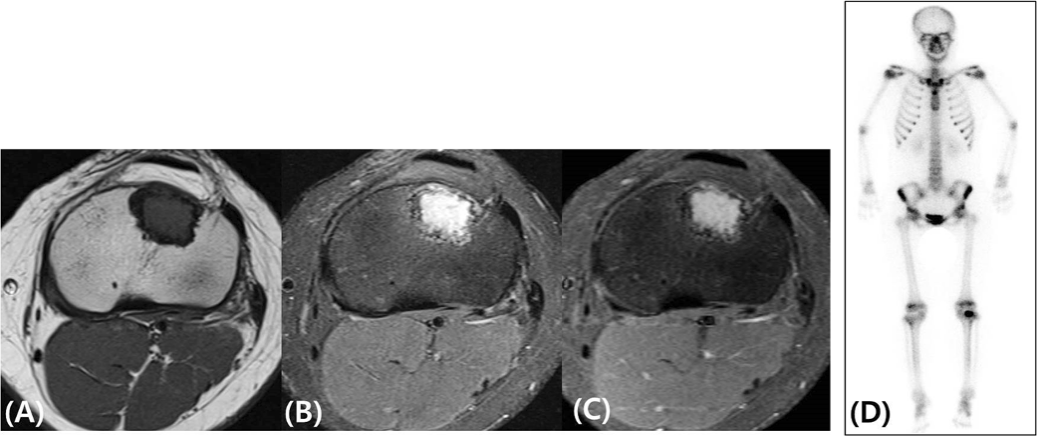
**Magnetic resonance imaging and scintigraphic images.** Magnetic resonance imaging scans show no peripheral bone edema and no fluid-fluid level. **(A)** T1-wieghted coronal magnetic resonance image (echo time/repetition time, 12/566ms) shows hypointensity of a proximal tibial lesion. **(B)** T2-weighted image (echo time/repetition time, 50/3660ms) shows hyperintensity. **(C)** Enhanced image (echo time/repetition time, 12/584ms) shows homogeneous enhancement. **(D)** Whole-body bone scan shows increased focal uptake only at the proximal epiphysis of the left tibia.

A 14-year-old Asian girl visited our hospital with radiographs taken 2 months earlier at a local clinic she attended for the evaluation of knee pain after slipping down. She had experienced no antecedent pain before slipping down, and the lesion was fortuitously found on radiographs. Anteroposterior and lateral radiographs were obtained. They showed a 1.5×2.0×2.0cm epiphyseal tumor in the proximal tibia. The matrix of the lesion had a clear, ground glass appearance, and the border showed well-defined marginal sclerosis. There was neither cortical disruption nor periosteal reaction. Computed tomography showed a sclerotic margin around the lesion, suggestive of a benign tumor (Figure 1). Magnetic resonance imaging (MRI) presented low signal intensity on a T1-weighted scan and high signal intensity on a fat-suppressed T2-weighted image, and homogeneous enhancement by gadolinium was seen in the lesion. On a technetium bone scintigraph, we detected focal, round, increased uptake only in left proximal tibia; otherwise, the image was unremarkable (Figure 2). There was no endocrinological abnormality such as precocious puberty, an absence of abnormal skin pigmentation and no specific family history. All the laboratory test results were within a normal range, except the aminotransferase level.

**Figure 3:**
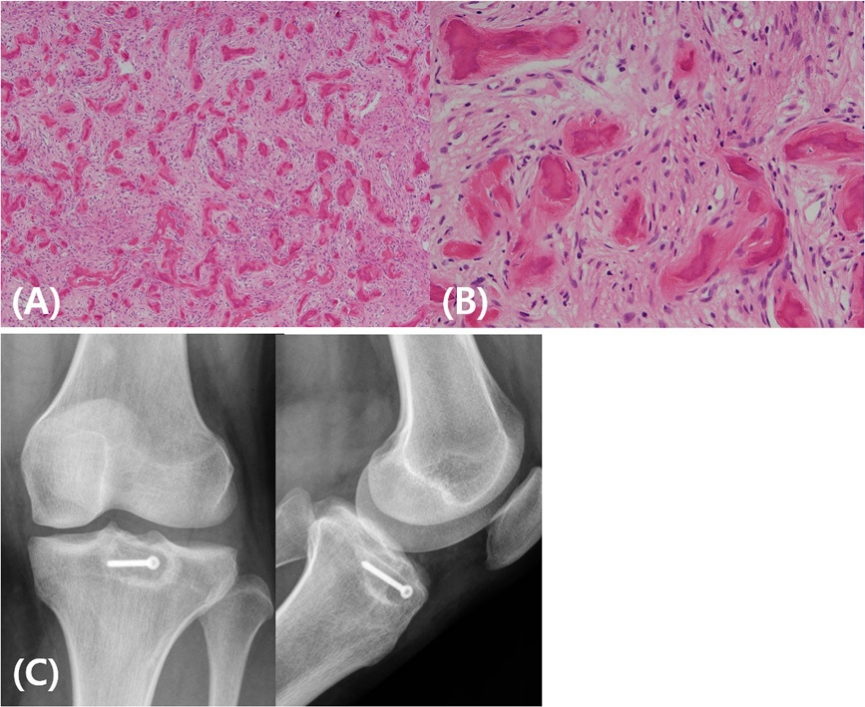
**Histopathologic findings and post-operative radiographs. (A)** Bone tissue is replaced by loose fibrous tissue with irregular spicules of immature bone (hematoxylin and eosin stain; original magnification, ×100). **(B)** The immature bone is formed from fibrous stroma without osteoblasts rimming (original magnification, ×400). **(C)** Simple radiographs obtained 2 years after surgery show bone formation with well-sustained screw fixation.

On the basis of the clinical and radiological findings, a benign bone tumor of a slow-growing nature, chondroblastoma, was suspected at first. Excisional biopsy, curettage and filling with demineralized bone matrix followed by 2.5mm screw fixation were performed. Histologic analysis of hematoxylin and eosin–stained specimens showed irregularly shaped spicules of immature bone without osteoblastic rimming and fibrous stroma without any mitotic activity. Radiographs taken 2 years after surgery demonstrated bone formation at the lesion, and the patient was able to squat and run freely without any pain (Figure 3).

## Discussion

FD is considered a common, benign skeletal disorder. Nixon and colleagues reported polyostotic cases and suggested that fibro-osseous aberration initially occurred in the epiphyseal growth plate with occasional extension into the epiphysis and metaphysis bidirectionally [6]. The mutation at the G_s_α gene after fertilization of somatic cells at chromosome 20q13.2-13.3 is regarded as the etiologic cause of FD. This mutation activates a cascade of the enzymatic reactions and results in incomplete differentiation of marrow stromal cells into abnormal osteoblasts [7]. Developmental failures in the process of primitive bone remodeling to mature into lamellar bone or failures in realignment due to mechanical stress are considered the pathophysiologic causes of the pain, deformity and pathologic factures [8].

Single-bone lesions of monostotic presentation without any other disturbance are the most common forms of FD, and monostotic FDs are known to enlarge in proportion to skeletal growth [9]. Polyostotic forms are less common and often continue to enlarge after complete skeletal maturation. This feature can cause progressive deformity and increased prevalence of pathologic fractures in polyostotic FD [8]. The most common skeletal deformity in FD is a discrepancy between the lengths of bilateral limbs, referred to as shepherd’s crook deformity of the proximal part of the femur [10].

Clinically, several imaging modalities are used routinely. In simple radiographs, FD presents as a well-marginated peripheral sclerotic bone lesion with a variety of patterns, which may be lucent, sclerotic or mixed or may have a ground glass appearance, depending on the amount of bone trabeculae, fibrous elements and calcification. Endosteal scalloping and focal cortical thinning may be present without cortical disruption or periosteal reaction [10–12]. Computed tomography, which is often not required, accurately delineates the extent of skeletal involvement and may be useful in evaluating craniofacial FD or lesions suspected to be sarcomatous transformations [12]. MRI can be used to differentiate low-grade central osteosarcomas when the radiographic features suggest FD [11]. In a pictorial review by Saha and co-workers, most of the FD was largely isointense with skeletal muscle on T1-weighted images. On T2-wieghted images, lesions are typically heterogeneously hyperintense with hypointense, isointense or markedly hyperintense areas within the lesion. In an enhanced MRI scan, FD may have patchy central enhancement, rim enhancement, homogeneous enhancement or any combination of these presentations. On both T1- and T2-weighted images, an outer hypointense rim is typically seen, and it corresponds with the sclerotic rim seen on radiographs [13]. During scintigraphic studies, FD typically exhibits markedly increased radionuclide accumulation in both early perfusion and delayed bone imaging. Even at low specificity, increased uptake in a delayed view is sensitive for early detection and assessment of the extent of involvement [14]. On the basis of radiographs, MRI scan and bone scan findings in our patient, chondroblastoma was considered the final diagnosis, not FD.

The histopathologic hallmark of the FD is fibrous tissue and immature, spindle-shaped, fibroblast-like cells within the bone marrow, and these fibrous tissues expand from the medullary cavity to the cortical bone. The strangely shaped trabeculae have been likened to "alphabet soup" or "Chinese characters." In our histologic results, immature bone filled with fibrous blastoma was found, and osteoblastic reaming was hardly seen. FD, which was not diagnosed pre-operatively, was our confirmatory diagnosis of our patient.

Chondroblastomas, subchondral cysts, infection-like Brodie abscesses and low-grade intramedullary central osteosarcomas can also occur in the epiphysis. Bone cysts may have more radiolucent lesions with thinner borders of lamellar bone and may show straw-colored fluid when aspirated. Chondroblastomas can be seen as subtle cartilaginous matrix on radiographs. They can present as marrow edema and joint effusion on MRI scans. Brodie abscesses can be displayed as serpiginous changes on radiographs and as the penumbra sign on MRI scans. Low-grade intramedullary central osteosarcoma is rare, but should be differentiated and may show the permeative border with a lack of a reactive shell, denser mineralization and more aggressive changes over time. Chondroblastomas, clear cell chondrosarcomas and Brodie abscesses are commonly painful [8, 15]. Our patient was completely asymptomatic prior to her slip and fall. In retrospect, if a computed tomography–guided biopsy or a simple bone biopsy had been performed prior to surgery, it might have prevented us from performing the extra operation.

## Conclusions

It is important to incorporate all the clinical, radiographic and pathologic features to diagnose monostotic FD, despite its unusual location in bone.

## Consent

Written informed consent was obtained from the patient’s legal guardian(s) for publication of this case report and any accompanying images. A copy of the written consent is available for review by the Editor-in-Chief of this journal.
